# HSP90 inhibitor AUY922 induces cell death by disruption of the Bcr-Abl, Jak2 and HSP90 signaling network complex in leukemia cells

**DOI:** 10.18632/genesandcancer.49

**Published:** 2015-01

**Authors:** Wenjing Tao, Sandip N. Chakraborty, Xiaohong Leng, Helen Ma, Ralph B. Arlinghaus

**Affiliations:** ^1^ Department of Translational Molecular Pathology, University of Texas M.D. Anderson Cancer Center, Houston, TX, USA

**Keywords:** HSP90, Bcr-Abl, CML, apoptosis, gel filtration

## Abstract

The Bcr-Abl protein is an important client protein of heat shock protein 90 (HSP90). We evaluated the inhibitory effects of the HSP90 ATPase inhibitor AUY922 on 32D mouse hematopoietic cells expressing wild-type Bcr-Abl (b3a2, 32Dp210) and mutant Bcr-Abl imatinib (IM)-resistant cell lines. Western blotting results of fractions from gel filtration column chromatography of 32Dp210 cells showed that HSP90 together with Bcr-Abl, Jak2 Stat3 and several other proteins co-eluted in peak column fractions of a high molecular weight network complex (HMWNC). Co-IP results showed that HSP90 directly bound to Bcr-Abl, Jak2, Stat 3 and Akt. The associations between HSP90 and Bcr-Abl or Bcr-Abl kinase domain mutants (T315I and E255K) were interrupted by AUY922 treatment. Tyrosine phosphorylation of Bcr-Abl showed a dose-dependent decrease in 32Dp210T315I following AUY922 treatment for 16h. AUY922 also markedly inhibited cell proliferation of both IM-sensitive 32Dp210 (IC_50_ =6 nM) and IM-resistant 32Dp210T315I cells (IC_50_ ≈6 nM) and human KBM-5R/KBM-7R cell lines (IC_50_ =50 nM). AUY922 caused significant G1 arrest in 32Dp210 cells but not in T315I or E255K cells. AUY922 efficiently induced apoptosis in 32Dp210 (IC_50_ =10 nM) and T315I or E255K lines with IC_50_ around 20 to 50 nM. Our results showed that Bcr-Abl and Jak2 form HMWNC with HSP90 in CML cells. Inhibition of HSP90 by AUY922 disrupted the structure of HMWNC, leading to Bcr-Abl degradation, nhibiting cell proliferation and inducing apoptosis. Thus, inhibition of HSP90 is a powerful way to inhibit not only IM-sensitive CML cells but also IM-resistant CML cells.

## INTRODUCTION

Chronic myeloid leukemia (CML) is a clonal of myeloproliferative neoplasm (MPN) resulting from the expansion of transformed primitive hematopoietic progenitor cells. The genetic hallmark of CML is chromosomal reciprocal translocation between chromosome 22 and chromosome 9 (t(9;22)(q34;q11)), leading to the generation of Philadelphia chromosome [1,2]. Part of the breakpoint cluster region (BCR) gene from chromosome 22 becomes fused to the second exon of c-ABL gene located in chromosome 9 to create BCR-ABL fusion gene. The resulting Bcr-Abl protein exhibits a constitutive tyrosine kinase activity caused by the disruption of N terminal of c-Abl self-inhibition sequence and the oligomerization of the Bcr-Abl protein catalyzed by the Bcr fusion. Cells transformed by Bcr-Abl acquire oncogenic ability, thereby transforming normal hematopoietic cells into leukemic cells. Importantly, Bcr-Abl in combination with cytokine receptors or growth hormone receptors mediates continuous activation of Jak2/Stats pathways [3-6].

Early stage CML patients are successfully treated with imatinib mesylate (IM). It inhibits kinase activities of both c-Abl and Bcr-Abl through competitive inhibition of binding of ATP to its docking site within kinase domain [7,8]. However, sustained remission by IM and other tyrosine kinase inhibitor (TKI) treatment becomes a challenge for TKI resistant CML patients [9,10]. The molecular mechanisms of IM resistance include: Bcr-Abl kinase domain mutations [11], overexpression of BCR-ABL protein [12], Lyn kinase overexpression and activation [13,14], alternative signal pathways via JAK-2/STAT-5 activation [15], up-regulation of protein kinase C η mediated c-Raf signaling pathway [16], existence of quiescent stem cells [17], intrinsic variability of enzymes in IM metabolism (e.g. cytochrome p450 system) [18], and increased levels of IM efflux transporters (e.g. ATP-binding cassette, sub-family B (MDR/TAP) and the multidrug resistant protein 1 (MDA-1))[19,20]. Thus, the limitations of TKI have resulted in the development of new targets and other therapeutic approaches in order to overcome the effect of resistance to TKI compounds.

Heat shock protein 90 (HSP90) is a ubiquitous molecular chaperone, which is associated with many different client proteins. HSP90 causes stabilization of client proteins, maintains their appropriate conformation and correct folding that is required for various events, such as signal transduction, cell cycle control and gene transcription [21,22]. Interfering the association between HSP90 and its client proteins by HSP90 inhibitors (e.g. 17-allylamino-17-demethoxygeldanamycin, 17-AAG) leads to the destabilization and degradation of its client proteins, resulting in cell death [23]. HSP90 is responsible for the chaperoning and maintenance of several oncogenic kinases such as Bcr-Abl, Raf and ErbB [5,21,24]. It affects the activity of client proteins critical for multiple steps in tumor progression, e.g. immortalization [25,26], reduction of apoptosis [27], angiogenesis [28] and invasion/metastasis[29]. HSP90 is up-regulated 10 fold in tumor cells suggesting its crucial role in maintaining tumor cells for growth and survival. Therefore, HSP90 has been chosen as a novel target for cancer therapy [24,30,31]. It has been reported that 17-AAG and IPI-504 (another HSP90 inhibitor) prolong survival of mice with wt Bcr-Abl or Bcr-AblT315I induced CML [32,33]. AUY922, another novel HSP90 inhibitor targeting ATPase activity of HSP90, exhibits significant activity against breast cancer [34] and decreases migration/invasion of lung carcinoma [35].

Bcr-Abl is the major driving force in CML and is considered to be the primary target for CML therapy. Bcr-Abl also maintains its stability and active tyrosine-kinase by inducing expression of SET through Jak2 [6] resulting in inhibition of the PP2A/Shp1 pathway [36]. Our previous study demonstrated that Bcr-Abl+ cells contain a high molecular weight Bcr-Abl/Jak2/HSP90 signaling network complex [37]. Disruption of the Bcr-Abl/Jak2/HSP90 complex by ON044580, a dual kinase inhibitor against Jak2 and c-Abl, resulted in Bcr-Abl protein degradation and apoptosis of CML cells.

In this study, we extended our previous findings [37] that Bcr-Abl+ cells carry a HMWNC with molecular size of 4-8 million Da which contains HSP90 and its client proteins including Bcr-Abl, Jak2, Lyn and Akt. Importantly, in these recent studies, we showed that the HSP90 ATPase inhibitor AUY922 efficiently disrupted the HMWNC resulting in the degradation of HSP90, Bcr-Abl, Jak2, Lyn and Akt. The association between HSP90 and Bcr-Abl (wt or mutant) was interrupted by AUY922 treatment. Tyrosine phosphorylation by the Bcr-AblT315I mutant showed a dose-dependent decrease following AUY922 treatment. AUY922 also significantly inhibited cell proliferation and induced apoptosis in both IM-resistant and sensitive Bcr-Abl+ cells. Our novel findings of CML cell death induced by disrupting Bcr-Abl/Jak2/HSP90 HMWNC through treatment with a HSP90 inhibitor reveals the critical role of HSP90 in stabilizing this HMWNC that maintains leukemic state. Our findings confirm that HSP90 is a promising therapeutic target for TKI-sensitive and -resistant CML patients.

## RESULTS

### HSP90 and its client proteins form HMWNC in Bcr-Abl positive cells

**Figure 1:**
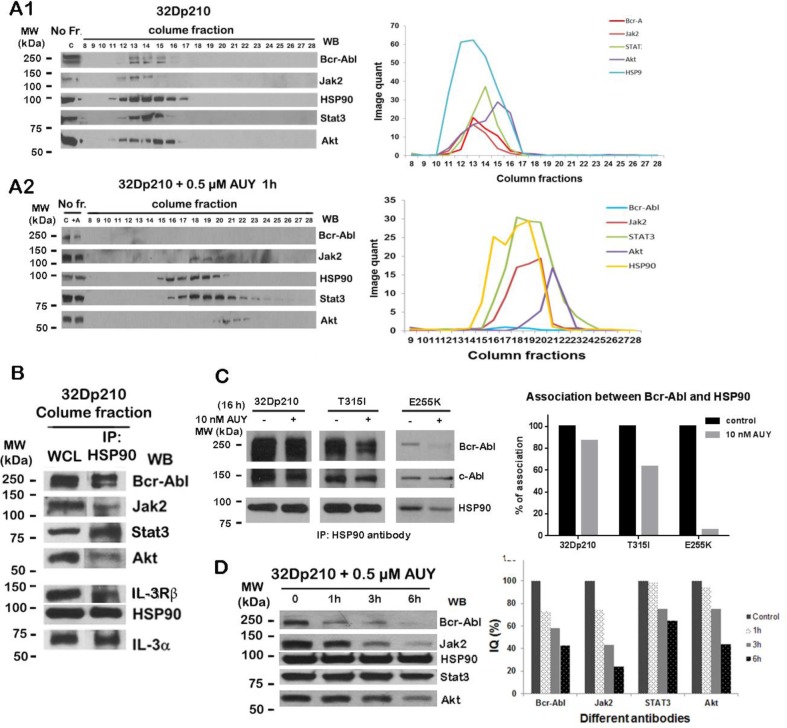
HMWNC is disrupted by HSP90 inhibitor AUY922 A1 and A2, HMWNC that was detected in Bcr-Abl+ cells was disrupted by AUY922 treatment. Proteins extraction of 32Dp210 cells treated with (A2 left panel) or without (A1 left panel) 0.5 μM AUY922 for 16 h were eluted from the gel filtration column. 25μl aliquot of each fraction was analyzed by Western blotting with indicated antibodies. No Fr, cell lysate without gel filtration. Right panel, image quantification of the Western blotting bands shown in left panel was graphically presented. B. Association of HSP90 and its client proteins in HMWNC. IP HSP90 column, the HMW region fractions from 32Dp210 cells were immunoprecipitated with HSP90 antibody following Western blotting with indicated antibodies. DWB, the same HMW region column fractions were directly Western blotted with the corresponding antibodies. C, The association between HSP90 and Bcr-Abl (wt, T315I or E255K) was impaired by AUY922. Left panel, cells were treated with 10 nM AUY922 for 16 h. Cell lysates were immunoprecipitated with HSP90 antibody followed by Western blotting with indicated antibodies. Right panel, the ratio of Bcr-Abl associated with HSP90. Protein levels were evaluated by image quantification. The levels of Bcr-Abl were normalized to HSP90 levels followed by normalization against non-treated controls to determine the association ratio, D. HSP90 client proteins level decreased under AUY922 treatment. Left panel, 32Dp210 cells were incubated with 0.5 μM AUY922 for 1, 3 and 6h. Cell lysates were analyzed by Western blotting with indicated antibodies. Right panel, the image quantification of protein bands normalized by HSP90.

We have reported the presence of the HMWNC of signaling molecules in Bcr-Abl+ cells [37], To further explore the components of the network complex in Bcr-Abl+ cells, lysates of 32Dp210 cell were fractioned by gel filtration column chromatography as previously described [37]. Fractions from the high molecular weight region (HMW) (fractions No. 8-17) and as well as the lower molecular weight region (LMW) (fractions No. 18-28) were analyzed by Western blotting. We found HSP90 and its client proteins including Bcr-Abl, Jak2, Stat3, and Akt were present in the same gel fractions of HMW (fraction No. 12-15), which were defined as the HMWNC with an estimated molecular weight of 2-6 million Dalton (Fig. 1A1, left panel). Image quantification of Western blotting bands showed that the gel column fractions containing highest levels of HSP90 and its client proteins (Bcr-Abl, Jak2, Stat3 and Akt) eluted in the same fractions of the column fractionation (Fig. 1A1, right panel). This suggests HSP90 and its client proteins are associated with each other in the HMWNC.

It has been reported that the members of the Bcr-Abl signaling pathways are linked to each other in CML cells [6,37,38]. Since Bcr-Abl regulates a variety of downstream proteins, we wanted to know whether other crucial signal molecules besides Bcr-Abl, Jak2 and HSP90 may be present in this HMWNC. To investigate this, the lysates of 32Dp210 cells were subjected to gel filtration. The fractions collected from both HMW and LMW were analyzed by Western blotting to identify various proteins. We used 32Dp210 cell lysates without gel filtration as a control. Western blot results were summarized in Table 1. Besides HSP90, Bcr-Abl and Jak2, additional proteins were detected in the HMWNC including Gab2, Gsk3ß, Erk1, Lyn, Ras, Raf, HIP, HOP and so on. These results indicated that the downstream signaling proteins regulated by Bcr-Abl are present in the HMWNC in 32Dp210 cells, suggesting that HSP90 client proteins (e.g. Bcr-Abl, Jak2) are protected from degradation by being chaperoned by HSP90 to form the HMWNC.

**Table 1:** Proteins that were identified in the high molecular weight region column fractions From the HMW (fraction No. 8-17) gel filtration fraction of 32Dp210 cell, an aliquot of 25 μl fraction were subjected to Western blotting to identify proteins by using specific antibodies.

HSP90 Client Proteins	Molecular Weight (kDa)
Bcr-Abl	210
Jak2	120
Stat3	85
Akt	60
Lyn	52/56
Erk1	42
Shc	56
Grb2	30
Ras	38
Gab2	98
Gsk3ß	46
IL-3 receptor α	70
IL-3 receptor β	120
HSP90	90
HOP	63
HIP	41
HSP70	70

### HSP90 associates with its client proteins in Bcr-Abl positive cells

Fig. 1A showed that HSP90 and its client proteins (e.g. Bcr-Abl, Jak2, Stat3, Akt) eluted in the same column fractions (fraction No. 12-15). To further investigate whether HSP90 directly binds to its client proteins, Co-IP experiments were performed with the gel filtration fractions collected from HMW (fractions no 12-15) of 32Dp210 cell lysate chromatography. We found the proteins present in HSP90 immune complex were Bcr-Abl, Jak2, Stat3, Akt, IL-3Rα and IL-3Rβ (Fig. 1B). The same column fractions directly Western blotted with corresponding antibodies (DWB) were used as a positive control for verification of each protein. These results indicate that HSP90 directly binds to the proteins within the high molecular weight network complex.

### AUY922 disrupts the HMWNC in Bcr-Abl+ cells

HSP90 is reported to be up-regulated in cancer cells to ensure the correct folding and function of the large quantities of oncoproteins and to protect them from ubiquitin proteasome-mediated protein degradation [21,30]. We predicted that, due to the CML disease, high levels of HSP90 are produced leading to the formation of the large network complex. Since we have detected a 2-6 million Dalton HMWNC composed of HSP90 and its client proteins in Bcr-Abl+ cells (Fig. 1A1), we wanted to know whether inhibition of HSP90 activity by the specific HSP90 inhibitor would disrupt this HMWNC and lead to client protein degradation. For this purpose, we incubated 32Dp210 cells with AUY922 (0.5 μM) for 1h followed by the gel filtration fractionation assay. Fractions from HMW and LMW were analyzed by Western blotting using various antibodies. Cell lysates of 32Dp210 cell treated with or without 0.5 μM AUY922 were used as controls. Fig. 1A2 showed that the levels of Bcr-Abl, Jak2, Stat3, and Akt significantly decreased after AUY922 treatment. Importantly, the peaks of eluted HSP90 and its client proteins were shifted to LMW (fraction No. 17-22) (Fig. 1A2). Since disruption of the Bcr-Abl/HSP90 complex causes degradation of Bcr-Abl [23,32,33,37], we could not detect Bcr-Abl protein in the fractions collected from either HMW or LMW. These results indicate that HSP90 inhibitor disrupted the HMWNC composed of signaling molecules in the Bcr-Abl driven pathways.

### The association between HSP90 and Bcr-Abl is disrupted by HSP90 inhibitor

One of the crucial functions of HSP90 is to chaperone proteins and protect them from degradation. Thus, we wanted to examine whether the interaction between Bcr-Abl and HSP90 was affected by treatment with AUY922. To determine this, 32Dp210, 32Dp210T315I and 32Dp210E255K cells were treated with 10 nM AUY for 16 h followed by Co-IP with HSP90 antibody. The Western blotting results of the Co-IP showed that wt Bcr-Abl and Bcr-Abl kinase domain mutants (T315I and E255K) were detected in HSP90 immune complex (Fig. 1C), indicating both wt Bcr-Abl and mutated Bcr-Abl were associated with HSP90. However, this association was disrupted when HSP90 activity was inhibited by AUY922 (Fig. 1C). The amounts of wt or mutated Bcr-Abl that associated with HSP90 were estimated by image quantification of protein bands in Western blotting (Fig. 1C, right panel). We also observed c-Abl bound to HSP90 but the level of c-Abl was only slightly reduced under AUY922 treatment, indicating c-Abl may be one of HSP90 client protein but it is less dependent on HSP90 for the stability. These Co-IP results confirmed our Western blot results of gel filtration eluents (Fig. 1B) that Bcr-Abl binds to HSP90 in HMWNC, suggesting that disruption of the association between HSP90 and Bcr-Abl by the HSP90 specific inhibitor AUY922 results in Bcr-Abl degradation.

### HSP90 inhibitor AUY922 reduces the levels of HSP90 client proteins in Bcr-Abl+ cells

Our present studies showed that HSP90 and its clients proteins (e.g. Bcr-Abl, Jak2, Stat3) form the 2-6 million Dalton HMWNC in Bcr-Abl + cells (Fig. 1A1), which was disrupted by AUY922 (Fig. 1A2). Next, we wanted to know whether disruption of the HMWNC by HSP90 inhibitor affects the stability of the HSP90 client proteins that are involved in Bcr-Abl driven signaling pathways. To address this question, 32Dp210 cells were treated with 0.5 μM AUY922 for 1, 3 and 6h. Western blotting results showed that the levels of Bcr-Abl and Jak2 dramatically decreased in a time-dependent manner following 0.5 μM AUY922 treatment (Fig. 1D). The levels of Stat3 and Akt did not change after 1h AUY922 treatment but started to decrease from 3h treatment. The level of Akt was greatly reduced under AUY922 treatment for 6h . The image quantifications of Western blotting bands of Stat3 and Akt (Fig. 1D right panel) showed reduction of protein levels. These results indicated that compared to Stat3 and Akt, the stabilization of Bcr-Abl and Jak2 are more dependent on HSP90, suggesting the crucial role of HSP90 in protecting Bcr-Abl and Jak2 from degradation in CML cells.

### AUY922 inhibits Bcr-Abl tyrosine phosphorylation and destabilizes Bcr-Abl protein

**Figure 2:**
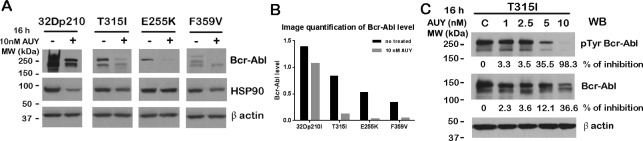
AUY922 down-regulated Bcr-Abl and its tyrosine phosphorylation A. 32Dp210, 32Dp210T315I, 32Dp210E255K and 32Dp210 F359V cells were treated with or without 10 nM AUY922 for 16h. Cell lysates were subjected for Western blotting with 8E9 and HSP90 antibody. B. The protein levels of Bcr-Abl (wt or mutants) were normalized by β actin and data were plotted in a histogram. C. 32Dp210T315I cells were treated with various doses of AUY922 for 16 h followed by Western blotting with indicated antibodies.

Next, we wanted to investigate whether wt Bcr-Abl cells (IM sensitive) or Bcr-Abl kinase domain mutant cells (IM resistant) responded differently to the treatment with the HSP90 inhibitor. To determine this, 32Dp210 and Bcr-Abl kinase domain mutant cells (T315I, E255K and F359V) were treated with 10 nM AUY922 for 16 h. Bcr-Abl protein levels were detected by Western blotting. As Fig. 2A and B showed, the total protein levels of mutated Bcr-Abl (T315I, E255K and F359V) dramatically decreased following AUY922 treatment whereas wt Bcr-Abl was only modestly decreased. HSP90 protein levels were only slightly reduced under the same treatment (Fig. 2A).

The tyrosine phosphorylation of Bcr-Abl is tightly related to its activity and function. Next we wanted to evaluate the Bcr-Abl kinase activity upon inhibition of HSP90 function. To study this, we treated IM-resistant 32Dp210T315I cells with various doses of AUY922 for 16h and examined total tyrosine phosphorylation levels of Bcr-AblT315I mutant. Interestingly, the level of Bcr-Abl T315I tyrosine phosphorylation was decreased by 35% by the presence of 5 nM AUY922, and was almost completely inhibited (~98%) by 10 nM AUY922 treatment (Fig. 2C). The Bcr-Abl total protein level as measured by 8E9 blotting did not greatly decrease. We only observed 36% inhibition on Bcr-AblT315I mutant by 10 nM AUY922 treatment (Fig. 2C).

### HSP90 inhibition interferes with the growth of wt Bcr-Abl cells and IM-resistant Bcr-Abl mutant cells

**Figure 3:**
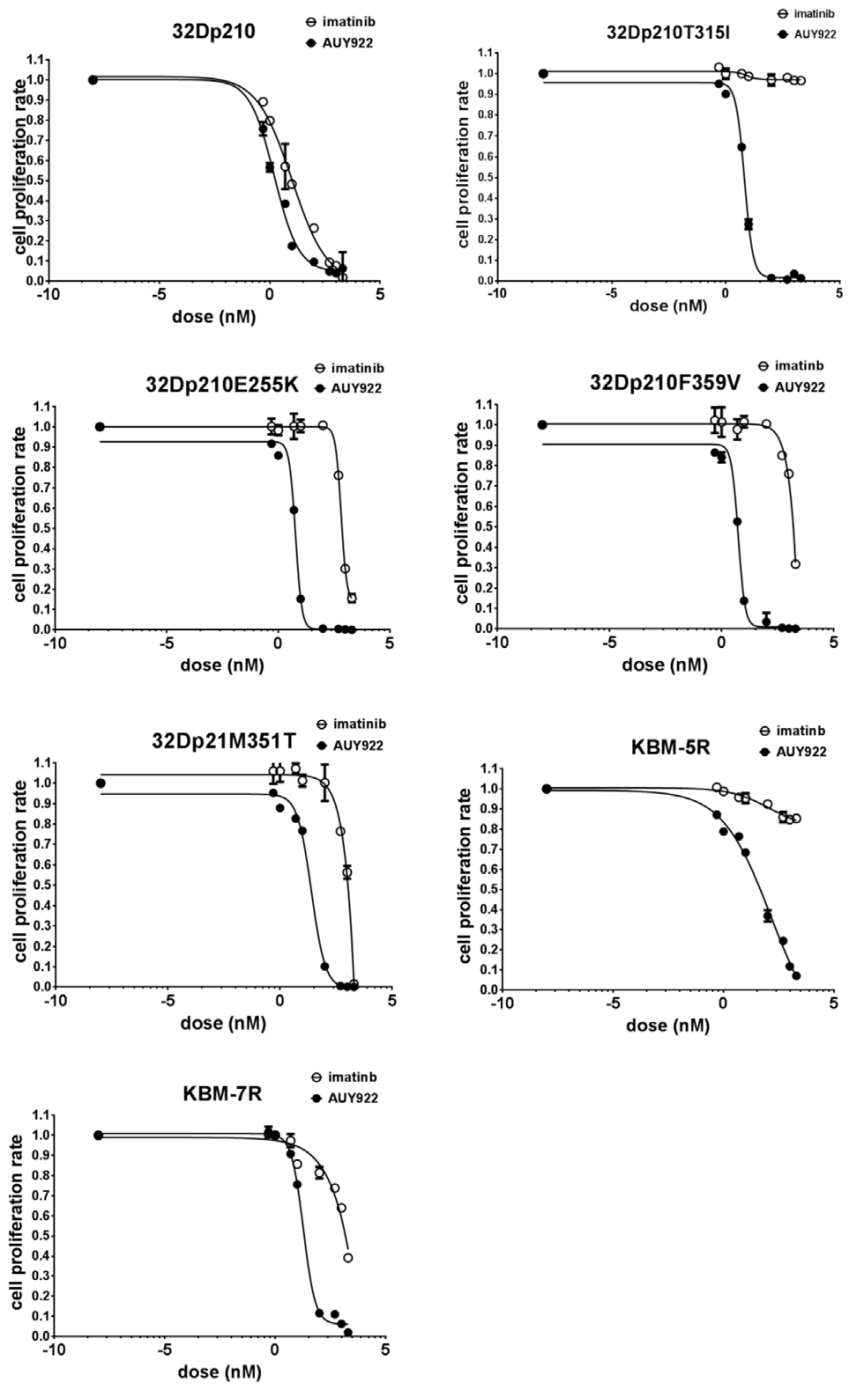
AUY922 inhibited cell proliferation in both IM-sensitive and -resistant CML cell lines Mouse leukemic cells and human CML cells were treated with various doses of either IM (5, 10, 100, 500, 1000, 2000 nM) or AUY922 (0.5, 1, 10, 100, 500, 1000 nM) for 72 h. Cell proliferation was measured by MTT assays.

Bcr-Abl protein stability is tightly related to the survival of CML cells. Thus, we were interested to know whether AUY922 inhibits the survival and proliferation of leukemic cells after disruption of HMWNC. To investigate this, various doses of AUY922 were applied to 32Dp210 cells or IM-resistant Bcr-Abl kinase domain mutant cells. MTT results showed that AUY922 strongly inhibited the proliferation of both IM-sensitive (32Dp210) and IM-resistant Bcr-Abl mutant mouse cell lines (such as 32Dp210T315I, 32Dp210F359V, 32Dp210E255K and 32Dp210M351T cells) and human CML cell lines (KBM-5R and KBM-7R) (Fig. 3). Compared to IM, AUY922 was much more effective for inhibition of cell proliferation of Bcr-Abl kinase domain mutant cells than wt Bcr-Abl cells (Table 2). The IC_50_ of AUY922 was more than 30 timeslower than the IC_50_ of IM in Bcr-Abl kinase domain mutant cells, but was only 0.5 times lower than that of IM (10 nM) for IM-sensitive 32Dp210 cells. Interestingly AUY922 strongly inhibited cell proliferation of 32Dp210T315I and KBM-5R cells (a human leukemic cells expressing Bcr-AblT315I mutant). The IC_50_ of AUY922 is 7 nM for 32Dp210T315I cells and 60 nM for KBM-5R cells, which were hundreds of times lower than that of IM (IC_50_ >10,000 nM) (Fig. 3 and Table 2). These results indicated that the Hsp90 inhibitor AUY922 efficiently down regulated cell proliferation of IM-sensitive as well as IM-resistant CML cells.

**Table 2:** IC_50_ of AUY922 and imatinib in wt Bcr-Abl and various Bcr-Abl kinase mutant cell lines Cell proliferation was analyzed by MTT assay. The 50% inhibition concentration values on cell proliferation (IC_50_) were calculated by fitting the data to a logistic curve

Cell line	IC_50_(nM)	
	Imatinib	NVP AUY922
32Dp210	10	6
32Dp210T315I (T315I)	>10,000	7
32Dp210F359V (E255K)	750	7
32Dp210E255K (F359V)	1500	7
32Dp210M351T (M351T)	1500	50
KBM-5R	>10,000	50
KBM-7R	1500	50

### HSP90 inhibitor caused G1 phase arrest in CML cells

**Figure 4:**
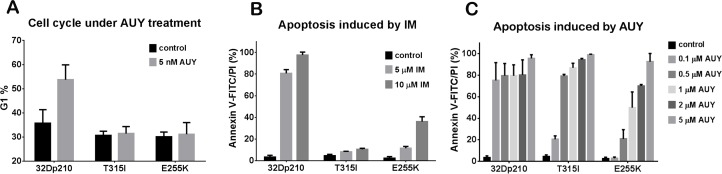
AUY922 caused cell cycle arrest in G1 phase and induced apoptosis in both IM-sensitive and resistant mouse leukemic cells A, The percentage of G1 phase cells from 32Dp210, 32Dp210T315I and 32Dp210E255K cells treated with 5 nM AUY922 for 16 h. B and C, Apoptosis of the same cells under 48 h treatment with either IM (5 and 10μM) (panel B) or AUY922 (10, 20, 50,100 and 200 nM) (panel C).

HSP90 is reported to be involved in cell cycle regulation by regulating cell cycle associated proteins [39,40]. Thus, we wanted to examine whether AUY922 affected the cell cycle. PI staining showed that an increase of 20% of 32Dp210 cells presented in G1 phase after 5nM AUY922 for 24 h (Fig. 4A), which indicated AUY922 induced G1 arrest in 32Dp210 cells. However, the cell cycle of IM-resistant 32Dp210T315I and 32Dp210E255K cells did not respond to AUY922, as G1 phase only slightly increased under the same treatment (Fig. 4A). We also didn't observe the changes in other cell cycle phases (e.g. G2 and S phase) of the IM-resistant cells after AUY treatment. These data showed that AUY922 caused G1 phase arrest in wt Bcr-Abl cells but had less effect on Bcr-Abl mutant cells, suggesting the regulation mechanism of cell cycle by kinase domain mutants of Bcr-Abl appears to differ from that of wt Bcr-Abl.

### AUY922 induced apoptosis in wt Bcr-Abl and Bcr-Abl mutant cells

We further investigated the effects of AUY922 on cell survival by apoptosis assays. IM-sensitive or -resistant Bcr-Abl+ 32D cell lines were treated with various doses of AUY922 or IM (5 and 10 μM) for 48h. As shown in Fig. 4B higher doses of IM (5 and 10 μM) induced more than 80% apoptosis in 32Dp210 cells but as expected had very less effect on 32Dp210T315I and 32Dp210E255K cells. Compared to IM, AUY922 significantly induced apoptosis in both wt Bcr-Abl and IM-resistant Bcr-Abl mutant cells (Fig. 4C). More than 70% apoptosis was observed in 32Dp210 cells under 10 nM AUY922, which was nearly 500 times lower than the dose of IM that achieved similar percentage of apoptosis. Furthermore, AUY922 efficiently induced apoptosis in IM-resistant Bcr-Abl mutant cells (Fig. 4C). The IC of AUY922 was less than 20 nM in 32Dp210 T315T cells and around 50 nM in 32Dp210E255K cells. These data indicate that AUY922 has high killing ability for both IM sensitive and resistant CML cells.

## DISCUSSION

In the present study, we investigated the biological function of the novel HSP90 inhibitor AUY922 in wt Bcr-Abl positive cell lines and IM-resistant cell lines. We have also elucidated the mechanism of action for induction of apoptosis in Bcr-Abl+ cells by HSP90 inhibitor AUY922. We showed that inside Bcr-Abl+ cells, HSP90 client proteins (e.g. Bcr-Abl and Jak2) exist as HMWNC with HSP90, and that client proteins directly interact with HSP90 (Fig. 1A1, 1B and 1C). As a key component of the HMWNC, HSP90 maintains the correct function of client proteins and protects them from degradation. Inhibition of HSP90 activity by AUY922 disrupted the structure of the HMWNC (Fig. 1A2), and decreased the interaction between HSP90 and Bcr-Abl (Fig. 1C), resulting in degradation of Bcr-Abl (Fig. 1D and 2A) and other client proteins (Fig. 1D). Treatment with AUY922 also down regulated the level of tyrosine phosphorylation of Bcr-Abl kinase domain mutant T315I (Fig. 2C). Compared to IM, AUY922 was more effective in inhibiting cell proliferation and inducing apoptosis in both IM-sensitive and resistant Bcr-Abl+ cell lines.

Our current gel filtration studies confirmed our previous finding of the existence of Bcr-Abl/Jak2/HSP90 network complex in Bcr-Abl+ cells (37). We further demonstrated that in 32Dp210 cells HSP90 and its client proteins (e.g. Bcr-Abl and Jak2) existed in the gel filtration column and eluted in same fractions of HMW (fraction No 11-16). This region represents a 2-6 million Dalton HMWNC (Fig. 1A1 and Table 1). Importantly, the co-migration of HSP90 and its client proteins in the HMWNC was disrupted by HSP90 inhibitor AUY922 (Fig. 1A2). Our data indicate inhibition of HSP90 activity leads to the destruction of HMWNC, suggesting an important role of HSP90 in maintaining this HMWNC in Bcr-Abl+ cells.

It is reported that HSP90 and other chaperone members provide a structural network for client proteins and maintain the biological function of several oncoproteins such as Bcr-Abl, Raf and ErbB2 [21,24,40]. We previously reported that Bcr-Abl is associated with various downstream proteins including Jak2, Grb2, Akt and GSK3ß to form HMWNC associated with HSP90 [5,37]. In the current study, the direct interaction between HSP90 and its client proteins (e.g. Bcr-Abl, Jak2, Stat3 and Akt) in the HMWNC was further investigated by Co-IP with HSP90 antibody, demonstrating various client proteins were bound to with HSP90 in the column eluent of the gel filtration column (Fig. 1B). Furthermore, association of HSP90 with Bcr-Abl and Bcr-Abl mutants (T315I and E255K) was significantly interrupted by AUY922 (Fig. 1C). We also found that the levels of HSP90 client proteins (e.g. Bcr-Abl, Jak2, Stat3 and Akt) were dramatically decreased in a time-dependent manner following 0.5 μM AUY922 treatment for up to 6 h (Fig. 1D). Since the main function of HSP90 is chaperoning its client proteins to maintain their correct post-translational conformation and activity [30,40], the mechanism of the client protein degradation may involve the disruption of the Bcr-Abl/Jak2/HSP90 HMWNC by AUY922 and the disruption of the individual HSP90-client protein interactions.

We have previously shown that Jak2 kinase activity was required for the stability of Bcr-Abl by phosphorylating Tyr 177 within the Bcr portion of Bcr-Abl [41], which triggers the binding of Grb2 to pTyr 177 leading Ras pathway members associate with Bcr-Abl [42]. As Shc is also known to bind to Bcr-Abl, the interaction between Grb2 and Shc also causes Ras pathway members to bind to a second site in Bcr-Abl besides the tyrosine 177 site [43] Our previous cellular based pull down experiments and the bimolecular fluorescence complementation studies documented that Jak2 directly binds to Bcr-Abl and c-Abl through their C-terminal and kinase domains of c-Abl [4,44]. Bcr-Abl and c-Abl tyrosine kinases specifically phosphorylated Y1007 of Jak2 leading to its kinase activation [4,41]. Jak2 activation is also required for tyrosine phosphorylation of Gab2 on YxxM which causes Gab2 binds to Grb2. The phosphorylated Gab2 regulates PI-3/Akt pathway leading to activation of c-Myc [5,45]. Furthermore, Jak2 interacts with Lyn and maintain Lyn kinase activity in CML cells through SET-PP2A-Shp1 pathway [6]. Bcr-Abl interacts with the IL-3 receptor ® subunit which is required for Bcr-Abl regulated Jak2 activation [46,47]. All these events suggest that Bcr-Abl functions as a scaffold for formation of the HMWNC.

IM is an effective drug used for the treatment of early stage CML patients. However, the IM resistance caused by the kinase domain mutations of Bcr-Abl (e.g. T315I and E255K) remains a challenge for the treatment of CML. Our Co-IP experiments demonstrated that Bcr-Abl kinase domain mutants (T315I and E255K) dissociate from HSP90 following the treatment of AUY922 (Fig. 1C). We further observed that AUY922 caused protein reduction of both wt Bcr-Abl and Bcr-Abl kinase domain mutants (T315I, E255K and F359V) (Fig. 2). Interestingly, 32D cells expressing Bcr-Abl kinase domain mutants are more sensitive to AUY922 treatment than wt Bcr-Abl. The total protein level of Bcr-Abl mutants (T315I, E255K and F359V) decreased by more than 5 times following AUY922 treatment, but the level of wt Bcr-Abl was only reduced by less than 2 times under the same treatment (Fig. 2A). As an indicator of Bcr-Abl function, the tyrosine phosphorylation of Bcr-Abl T315I mutant was dramatically inhibited by 98% in the presence of 10 nM AUY922 (Fig.2C).

As the result of Bcr-Abl protein degradation, cell proliferation and survival were down-regulated by AUY922. As shown in Table 2 and Fig. 3, AUY922 efficiently suppressed cell growth of both IM-sensitive and -resistant cell lines, especially 32Dp210T315I and KBM-5R with IC_50_ around 7 nM and 50 nM, respectively, which were more than 200 times lower than that of IM. Furthermore, AUY922 significantly induced apoptosis in 32Dp210T315I and 32Dp210E255K cells which are resistant to IM (Fig. 4C). However, the effects on cell cycle of Bcr-Abl mutant cells were less responsive to AUY922 treatment compared to wt Bcr-Abl+ cells, indicating different roles of HSP90 in regulating cell cycle of wt Bcr-Abl and Bcr-Abl mutant cells.

In summary, we document the important role of HSP90 in chaperoning its client proteins (e.g. Bcr-Abl, Jak2) in part by maintaining the structure of the HMWNC that is important for Bcr-Abl signaling and the survival of Bcr-Abl+ cells. Disruption of this HMWNC and the interaction between the individual HSP90 client protein by low concentrations of the HSP90 inhibitor AUY922, resulted in the degradation of client proteins and cell death. Thus, compared to traditional strategies of treating CML patients with TKIs, targeting HSP90 with its inhibitors, which we found to be a key factor in maintaining the structure of the HMWNC, may be a promising therapeutic approach against IM-sensitive and -resistant CML.

## METHODS

### Reagents and antibodies

The-AUY922 compound was kindly provided by Novartis institution for BioMedical Research. Imatinib was purchased from LC laboratories. Imatinib was diluted in PBS and AUY922 was dissolved in 100% DMSO (dimethyl sulfoxide) at the stocking concentration of 10 mM, and was aliquoted into microtubes to be stored at −20°C until use. Commercially available antibodies used were anti-phospho-Jak2Y1007 (Mollipore, Cat# 04-1098), Stat3 (Cell signaling, Cat# 9132), Akt (Cell signaling, Cat# 9272), HSP90 (Cell signaling, Cat# 4874), c-Abl (Cell Signaling, Cat# 2862), phosphotyrosine (4G10) (Millipore, Cat# 05-321), alpha tubulin (B-7) (Santa Cruz Biotechnology, Cat# sc-5286), beta actin (N-21) (Santa Cruz Biotechnology, Cat# sc-130656). Sepharose beads conjugated Jak2 antibody was purchase from Cell Signaling (Cat# 4089). Anti-Abl SH2 domain monoclonal antibody (8E9) was produced by our own lab.

### Cell culture

32Dp210 cells (wt Bcr-Abl+ 32D cells) sensitive to IM were generated by retroviral transfection of 32D mouse myeloid cells with MigR-1Bcr-Abl p210 (b3a2) construct. 32D cells expressing Bcr-Abl kinase domain mutants that were resistant to IM were kindly provided by Dr E. Premkumar Reddy (Icahn School of Medicine at Mount Sinai, New York). IM resistant human leukemic cells KBM-5R and KBM-7R were kindly provided by Dr Miloslav Beran (the University of Texas M.D. Anderson Cancer Center). All cells were maintained in standard RPMI 1640 medium supplemented with 10% fetal bovine serum (FBS), 100mg/L pennicilin/streptomycin, and 2 mM Glutamine (Gibco/BRL). Cells were cultured at 37oC in a humidified 5% CO_2_

### Gel filtration column chromatography

Gel filtration column chromatography was done as previously described [37]. Briefly, the dimension of the column was 50cm length x 0.7cm diameter (Econo column, Bio-Rad, Hercules, CA, Cat#737-0752), and column material used in this study was Superose 6 prep grade gel filtration (Amersham-Biosiences, GE Healthcare, Piscataway, NJ, Cat#17-0489-01). The composition of the elution buffer was 30 mM HEPES (pH 7.4) containing 150mM NaCl, 10% glycerol, 0.5% NP-40 and the elution rate was 4.56 ml/h. Cells were lysed as described [4]. Cell lysates were loaded onto the surface of Superose 6 gel filtration column. Cell lysates were eluted from the column using the elution buffer and the fractions (500 μl) were collected in microfuge tubes in a fraction collector. The whole procedure was conducted at 4°C. The size of Bcr-Abl/Jak2/HSP90 protein network complex was estimated to be between 2 and 6 million Da.

### Immunoprecipitation and western blotting

Whole cells lysates were prepared in 1% NP-40 lysis buffer containing a cocktail of protease and phosphatase inhibitors (Thermo Scientific, Cat# PI-78442) as the standard procedure. Co-immunoprecipitation and western blotting were as described previously [4]. The levels of protein expression were quantified by image J software. Data were analyzed by Microsoft office Excel2010 and plotted into histograms by Graphpad Prism 6 software.

### Cell proliferation via MTT assay

Cell viability/proliferation was performed as described [48]. Briefly, after being washed twice with PBS, cells were re-suspended in RPMI medium and were seeded in 96 well plate at 10,000 cells per well. Cells were incubated with either serial dilutions of AUY922 (0.5-1000 nM) or IM (5-2000 nM) prepared in cell growth medium at 37oC for 72 hours. Cell proliferation was assessed by premixed WST-1 cell proliferation reagent (Clontech, Cat# 630118) according to the instruction of the manufacturer. Control samples contained the respective concentration of DMSO. The quantity of dye-stained cells was directly related to the number of metabolically active cells. The absorbance was measured on a microtiter plate reader at a wave length of 420-480 nm. Data from three independent experiments were averaged and normalized against non-treated controls to generate the dose-response curve by Graphpad Prism 6 software. The 50% inhibition concentration (IC_50_) values were calculated by fitting the data to a logistic curve.

### Cell cycle assay

Cells cycle analysis was performed by the standard propidium iodide staining. Cells were washed with PBS twice and seeded at 0.2×10^6^ cells per each well of 12 well plates. AUY922 compound was added at the final dosage of 5 nM and cell cycle was analyzed 24h after treatment. Cells were harvested and fixed in PBS/70% ethanol at 4 oC overnight. Fixed cells were re-suspended in the staining solution containing 40 g/ml propidium iodide (Sigma-Aldrich, Cat# 81845), 100 g/ml RNase A, following incubation at 37oC for 30 min and analyzed by flow cytometry (Beckman coulter). The percentage of cells in G1 phase was measured by WinMDI 2.8 software for cell cycle analysis of DNA histograms. Data from three independent experiments were averaged and put into Graphpad Prism 6 software to generate histogram.

### Apoptosis assay

Apoptosis assay was performed as described previously [44]. Briefly, cells were seeded in 12 well plate at 0.5×10^6^ cells per well. HSP90 inhibitor AUY922 or imatinib was added to each well to achieve the desired dose. After 48 h treatment, cells were stained by Annexin V-FITC as part of the apoptosis detection kit (BD Pharmingen Cat# 556547) and apoptosis was analyzed by flow cytometer (Beckman coulter). Each treatment was done in triplicate. The apoptosis data from three independent experiments were averaged and plotted by Graphpad Prism 6 software.
